# Comparative Performance of Electrochemiluminescence Immunoassay and EIA for HIV Screening in a Multiethnic Region of China

**DOI:** 10.1371/journal.pone.0048162

**Published:** 2012-10-29

**Authors:** Xiaohui Bi, Hongxia Ning, Tingting Wang, Dongdong Li, Yongming Liu, Tingfu Yang, Jiansheng Yu, Chuanmin Tao

**Affiliations:** 1 Division of Clinical Microbiology, West China Hospital, Sichuan University, Chengdu, China; 2 Department of Laboratory Medicine, West China Hospital, Sichuan University, Chengdu, China; Rollins School of Public Health, Emory University, United States of America

## Abstract

**Background:**

The recent approval of 4th generation HIV tests has forced many laboratories to decide whether to shift from 3rd to these tests. There are limited published studies on the comparative evaluation of these two different assays. We compare the performance of fourth-generation electrochemiluminescence immunoassay (ChIA) and third-generation enzyme linked immunosorbent assay (EIA) for human immunodeficiency virus (HIV) screening and gauge whether the shift from EIA to ChIA could be better in a multiethnic region of China.

**Methodology/Principal Findings:**

We identified a large number of routine specimens (345,492) using two different assays from Jan 2008 to Aug 2011 in a teaching hospital with high sample throughput. Of the 344,596 specimens with interpretable HIV test results, 526(0.23%) of 228,761 using EIA and 303(0.26%) of 115,835 using ChIA were HIV-1 positive. The false-positive rate of EIA was lower than that of ChIA [0.03% vs. 0.08%, odds ratio 0.33 (95% confidence interval 0.24, 0.45)]. The positive predictive value (PPV) of EIA (89.6%) was significantly higher than that of ChIA (76.1%) (<0.001), reflecting the difference between the two assays. The clinical sensitivities of two assays in this study were 99.64% for EIA and 99.88% for ChIA.

**Conclusion:**

Caution is needed before shifting from 3rd to 4th generation HIV tests. Since none of these tests are perfect, different geographic and ethnic area probably require different considerations with regard to HIV testing methods, taking into account the local conditions.

## Introduction

Acquired Immunodeficiency Syndrome (AIDS) remains the most devastating outcome of HIV infection. It has had a profound effect on human illness and death over the last 30 years and has close to a 100% fatality rate in untreated patients. The laboratory plays a key role in preventing the spread of this epidemic and laboratory-based methods have undergone tremendous change. Universal screening to identify HIV infection is recommended so that infected individuals can be linked to care, antiretroviral medications and prophylaxis against opportunistic infections. Since the first HIV test was introduced, the performances of HIV screening assays have improved continuously. Selection of a particular test method for HIV screening should be based on objective measures of performance. Although there are several manners of evaluating test performance [1], clinicians usually make judgments about tests by interpreting sensitivity (Se) and specificity (Sp). Third-generation assays using an antigen–antibody–antigen sandwich technique, have led to a big improvement in Se and Sp, and have, until recently, been the method of screening in most centers of China. Fourth-generation ChIAs combine detection of HIV antibodies with detection of HIV antigens. The recent approval of 4th generation HIV tests in China has forced many laboratories to weigh the costs and benefits of transitioning to these tests; therefore, the comparison of 3rd and 4th generation HIV tests is a topic of current relevance and importance. With the chemiluminescent analyzer, 4th assays can offer more rapid turnaround time. Besides, it is P24 antigen of 4th assays that make early screening possible. However, clinicians and laboratorians are asked to how to best interpret the discordant results due to the high false-positive results.

In China, it is one of the best ways, in fact maybe the only way to find HIV infection with routine screening in hospitals and blood stations. The hub of the western China, Chengdu, is also a multiethnic region. Therefore, the clinical diagnosis performance is of great importance for HIV screening in this multiethnic region of China. As the setting, standard or patient group changes, prevalence and performance may change [2]. The same HIV test addressed in different studies frequently displays considerable variation in the results [3]. Se and Sp are just test properties that describe the behavior of the test in a particular situation. Some of this variability is due to chance as many diagnostic studies have small sample sizes [4]. Prevalence of HIV infection may also influence observed test accuracy [5]. We should take into account the prevalence of disease in order to properly use and interpret diagnostic tests [6], [7]. Minimum accuracy of HIV diagnostic tests is considered the pillar on which testing strategies for all settings must be based. Systematic reviews and meta-analyses have shown that performance of the same test in different settings may vary according to several factors [8], [9], such as the reference standard or the “gold standard”, clinical spectrum bias, role of the prevalence of the disease, operational characteristics. Choosing the appropriate serological screening algorithms in geographical areas with multiethnic communities require identification of assays that have optimal Se and Sp for that particular setting [3]. In addition, the complexity and feasibility of these protocols need to be addressed. It is important to evaluate periodically and select optimally performing serological assays before their use in large scale. Choice of an ideal, cost-effective, and rapid test for HIV infection is of immense value for use in developing countries like China, where resources are limited. 345,492 HIV test results were evaluated from laboratories operated by a largest hospital under routine conditions. We compare the performance of ChIA and EIA for HIV screening and gauge whether the shift from EIA to ChIA could be better in a multiethnic region of china with high sample throughput.

## Materials and Methods

### Ethics Statement

The observational study received ethical approval from West China Hospital of Sichuan University.

The study was based on existing laboratory data (N = 345492) collected at a largest hospital laboratory. The data were analyzed anonymously.

### Study Population and Screening Systems

**Figure 1:**
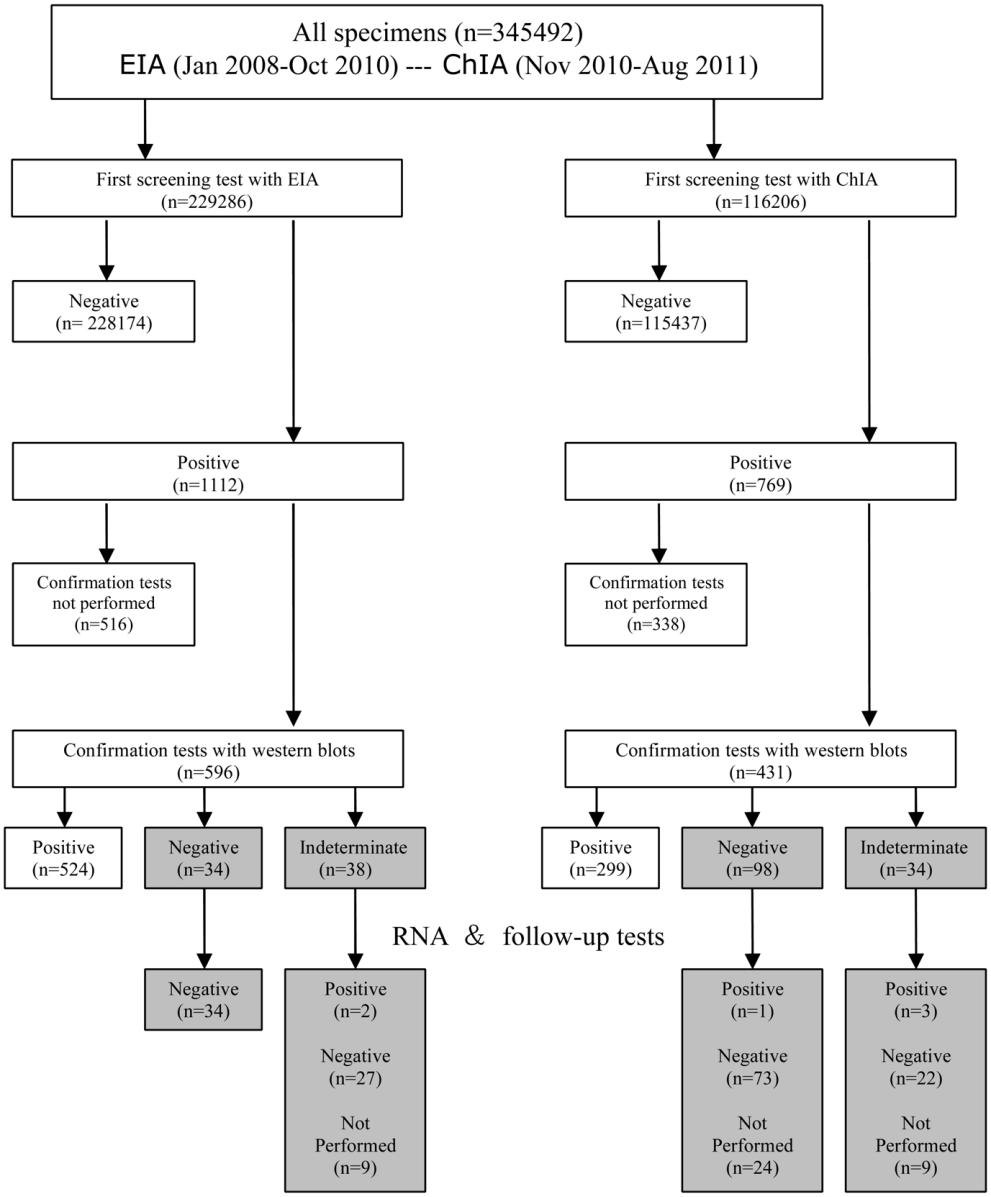
The operational flow chart and comparison of the results obtained by two HIV testing assays.

Two fully automated analyzers were compared, the Elecsys HIVcombi assay (ChIA) on the cobas e 601 and MODULAR E170 analyzers (Roche Diagnostics, Mannheim, Germany) and Anti-HIV ELISA (EIA) kit ZHUHAI LIVZON on TECAN (freedom evolyzer, Switzerland). We retrospectively collected testing data without personal identifiers from serum or plasma specimens from West China Hospital (a university hospital with 4300 beds) in a multiethnic region of China from Jan 2008, through Aug 2011. Until Oct 2010, screening for HIV infection was carried out with a third generation Ab assay (Anti-HIV ELISA kit ZHUHAI LIVZON). Since Nov 2010, a fourth-generation immunoassay (Elecsys HIVCOMBI assay on the Cobas analyzers) has been used at our laboratory for both diagnosis and screening purposes. Therefore, different samples were tested by the ChIA and the EIA in different period (Figure 1). They were conducted according to manufacturer’s instructions.

The result of a sample is given as in the form of a cutoff index (COI) for ChIA or ratios of specimen signals to the cutoff values (S/CO) for EIA (not light units for ChIA's or absorbance for EIA's), where the value greater than or equal to 1.00 is considered reactive.

### Study Design and Procedures

We confirmed HIV infectious status with the following algorithm. Specimens with initial HIV-negative results were considered uninfected. Any reactive result on a screening test is retested in duplicate. If both of the tests are reactive, the specimen is reported as repeated reactive and is submitted for confirmation testing. Confirmatory tests include Western Blot (WB) and HIV-RNA tests. If the WB testing is negative or indeterminate, nucleic acid tests (NAT) for viral RNA would be suggested to detect an early infection. Follow-up specimens were also used for resolution of discordant results as described in Figure 1. The procedure of follow-up tests are absolute same as initial tests, including screening and confirmation tests. In our hospital, we use real-name registration system. The ID value stored in this list for each patient is a unique identification. That unique ID is not only the best way to identify each member, but also one way to guard privacy. In this way, we were able to link initial test results with follow-up results.

### The Confirmatory Tests

The confirmatory tests include Western Blot HIV blot 2.2(MP Diagnostics, Singapore) and COBAS AmpliPrep/COBAS TapMan HIV-1 Test (Roche Diagnostics).

In China, HIV-1 western blots are usually interpreted following the National Guideline for Detection of HIV/AIDS (2009 edition), which require detection gp41 and gp120/160 (p24 and gp41/gp120/gp160) for positive results. The absence of all bands is a negative result. HIV-associated bands that are present but do not meet the criteria for positivity are an indeterminate pattern. HIV results of individual samples were recorded after reading by two different laboratory technologists and according to manufacturer’s criteria for interpretation of positive, negative or inconclusive results. Infection with HIV-2 is rare in our setting, and specific testing for HIV-2 was not performed as part of this study.

HIV-1 DNA PCR (COBAS AmpliPrep/COBAS TapMan HIV-1 Test, Roche Diagnostics) was used to test samples with western blot-discordant results as previously described. The features of this assay can be found in Supplementary Materials S1.

### Statistical Analysis

Most of the data were from our clinical laboratory information system (LIS). Some follow-up data were searched from Hospital Information System (HIS). The statistical software package SPSS 11.0 (SPSS Inc, Chicago, USA) was used for data analysis. Statistical comparisons were made using the chi-square test to assess the difference between two proportions. Tests of statistical significance included the 95% confidence intervals of unadjusted relative risks. values of less than 0.05 were considered statistically significant. The data were reviewed by laboratory quality control staff, and data entry was performed by two independent persons.

## Results

### Clinical Specificity, FPR, PPV

During the analysis period, of those 345,492 specimens, 116,206(33.6%) were carried out with EIA, 229,286(66.4%) with ChIA (Figure 1). Of the 344,596 specimens with interpretable HIV test results, 526(0.23%) of 228,761 using EIA and 303(0.26%) of 115,835 using ChIA were HIV-1 positive. The false-positive rate of EIA was lower than that of ChIA [0.03% vs. 0.08%, odds ratio 0.33 (95% confidence interval 0.24, 0.45)]. The positive predictive value (PPV) of EIA (89.6%) was significantly higher than that of ChIA (76.1%) (<0.001), reflecting the difference between the two assays (Table 1).

**Table 1:** Data on the performance of a third-generation HIV immunoassay and a fourth-generation HIV Ag/Ab combined assay in the routine screening for HIV infection.

Assay	Period of time	TP	FP	TN	False positive rate % (95% CI)	PPV% (95% CI)
EIA	Jan 2008–Oct 2010	526	61	228174	0.03(0.02,0.04)	89.6(87.1,92.1)
ChIA	Nov 2010–Aug 2011	303	95	115437	0.08(0.06,0.10)	76.1(71.9,80.3)

TP = true-positive, FP = false-positive, TN = true-negative, PPV = positive predictive value, CI = confidence interval.

Besides, when switching from EIA to ChIA, there were paired sample testing done to validate the method prior to implementation. 3740 routine samples were subjected to parallel testing on each assay. The results of 2×2 table are shown in Table 2. The clinical specificity and PPV of two assays are 99.97%, 91.7% for EIA and 99.91%, 78.6% for ChIA.

**Table 2:** PPV, Clinical specificity and Kappa of EIA and ChIA Assays in 3740 samples with parallel testing on each assay.

Assay	TP	FN	TN	FP	PPV% (95% CI)	Clinical specificity% (95% CI)	Kappa index
EIA	11	0	3728	1	91.7 (90.03,93.29)	99.97 (99.63,1)	0.896
ChIA	11	0	3726	3	78.6 (77.3, 79.9)	99.91 (99.57,1)	0.882

TP = true-positive, FN = false-negative, TN = true-negative, FP = false-positive, PPV = positive predictive value, CI = confidence interval.

### Clinical Sensitivity

Of 829 HIV-1 infected persons from various stages of disease during the analysis period, 826(828) were found to be reactive with EIA (ChIA). The clinical sensitivities of two assays in this study were 99.64% for EIA and 99.88% for ChIA (Table 3).

**Table 3:** Clinical sensitivities of assays in 829 HIV-1 infected persons from various stages of disease.

Assays	N	Reactive	Clinical sensitivity(95% lower CI)
EIA	829	826	99.64% (99.41%)
ChIA	829	828	99.88% (99.64%)

CI = confidence interval.

### Precision and Kappa Index

The precision was evaluated by using a modified form of the EP5-A2 protocol of the Clinical and Laboratory Standards Institute [10]. Experiments were performed 6 times a day for 10 days with human serum and quality control materials recommended by each manufacturer. Precision results are shown in Table 4. Within-run and between-run assay percent coefficient of variation values for positive controls ranged from 1.3% to 2.7% and 2.7% to 4.6%, respectively. Due to low mean values for the negative control, Within-run and between-run assay percent coefficient of variation values for negative controls ranged from 3.6% to 6.7% and 8.3% to 10.9%, respectively.

**Table 4:** Within-run and between-run assay precision results of EIA and ChIA.

	EIA						ChIA						
	Repeatablity			Intermediate precision			Repeatablity				Intermediate precision		
Sample	MeanS/CO	SD S/CO	CV %	Mean S/CO	SD S/CO	CV %	MeanCOI	SD COI	CV %	MeanCOI		SD COI	CV %
HS, negative	0.153	0.007	4.3	0.149	0.016	10.9	0.327	0.01	3.6	0.413		0.043	10.3
HS, positive	14.5	0.392	2.7	14.9	0.685	4.6	69.3	1.66	2.4	70.2		2.67	3.8
PC HIV 1	0.147	0.01	6.7	0.133	0.012	8.7	0.232	0.014	6.1	0.253		0.021	8.3
PC HIV 2	3.7	0.081	2.2	3.9	0.14	3.6	12.5	0.2	1.6	12.3		0.332	2.7
PC HIV 3	4.1	0.074	1.8	4.6	0.17	3.7	15.6	0.203	1.3	14.8		0.429	2.9

Repeatablity = within-run precision, Intermediate precision = between-run, HS = human serum, PC = PreciControl, SD = standard deviation of s/co or COI ratio, CV = coefficient of variation of s/co or COI ratio.

The calculation of Kappa index is based on the difference between how much agreement is actually present (“observed” agreement) compared to how much agreement would be expected to be present by chance alone (“expected” agreement). The data layout is shown in Table 2. Generally, a kappa of 0.896 or 0.882 is in the “perfect” agreement range between two observers [11]. They are also calculated basing on 3740 paired samples with parallel testing on each assay.

### Follow-up Results

162 western blot-discordant samples were followed up by repeat RNA tests and WB testing of subsequent samples from the same patients over a 3–12 month period. 156 samples gave negative results. Mean time from screening to follow-up on specimens that did not become positive is 8 weeks. Only 6 samples not initially meeting criteria for positivity turned clear-cut positive (Figure 1, Table 5). The seroconversion time and changes of follow-up tests on these 6 samples were shown in Table 6.

**Table 5:** Follow-up of 162 Western blot-discordant samples.

	EIA	ChIA
IND→POS	2	3
IND→NEG	27	22
NEG→POS	0	1
NEG→NEG	34	73

POS = positive, NEG = negative, IND = indeterminate.

**Table 6:** Six western blot-discordant samples that had seroconverted to HIV-positive status.

Time to seroconversion (days)	Changes of Western Blot Pattern or HIV-1 RNA tests
28	gp160→p24,p31,p66,gp41,gp120,gp160
31	gp160→p17,p24,p31,p66,gp41,gp120,gp160
39	p24→p17,p24,p31,p51,p66,gp41,gp120,gp160
45	WB bands absent→HIV-1 RNA positive
63	p24→p17,p24,p31,p51,p55,p66,gp41,gp120,gp160
98	gp160→p17,p24,p31,p51,p66,gp41,gp120,gp160

### Confirmation Results in Relation to COI(s/co) Ratio

We investigated (COI) s/co ratios of screening assays according to HIV infection status. As mentioned above, HIV infection status was determined by WB, HIV-1 RNA and follow-up tests. The number of cases with HIV infection increased in relation to the s/co (COI) ratio (Table 7). Most weakly reactive or borderline results were negative when determined by WB (western blot) and RNA tests. In cases with COI ratio (ChIA) <15.0, there was no positive result by confirmatory tests. In the ChIA assay, only 2 (2.2%) of 89 cases with COI ratio of <50 had HIV infection.

**Table 7:** Confirmation results in relation to COI(s/co) ratio.

COI(s/co) ratio		No. of cases in each group	No. (%) of cases	
			Positive	Negative
ChIA (COI)				
	<15	77	0(0.0)	77(100.0)
	15–50	12	2(16.7)	10(83.3)
	50–150	32	27(84.4)	5(15.6)
	150–200	30	29(96.7)	1(3.3)
	200–400	143	141(98.6)	2(1.4)
	400–800	91	91(100.0)	0(0.0)
	>800	13	13(100.0)	0(0.0)
Total		398	303(76.1)	95(23.9)
EIA (s/co)				
	<2	24	1(4.2)	23(95.8)
	2–4	18	5(27.8)	13(72.2)
	4–6	31	16(51.6)	15(48.4)
	6–8	29	28(96.6)	1(3.4)
	8–10	60	58(96.7)	2(3.3)
	10–20	341	337(98.8)	4(1.2)
	>20	84	83(98.8)	1(1.2)
Total		587	526(89.6)	61(10.4)

## Discussion

This study is a first attempt to compare the two assays in the context of highly complex and multiethnic region of China. Our results indicate that the performance of both assays was satisfactory in this setting. In a publication about comparison of tests, the authors concluded that differences between two tests of less than 1.3% in Se and 1.4% in Sp were not statistically significant for that dataset [3]. The most relevant issue is not small differences in accuracy of available HIV diagnostic tests [12], [13], but the characteristics of the population subgroup in which the test will be applied. Although information on performance can help clinicians make decisions about tests, good diagnostic performance is a necessary but not sufficient condition for the effectiveness of a test [4]. At the same time, meta-analysis has been increasingly used to assess diagnostic methods and it is of the utmost importance that the full-scale evaluation of any assay combinations in the context in which they will be used be performed before wide scale implementation [14], [15].

As shown in Table 1, the false positive rate of repeatedly reactive sample in ChIA is 0.08%, which seemed nearly 3 times higher than that of EIA, as the former combine two reactions of a different nature, which may require different cut-off thresholds for optimal Sp and Se in a single test [16], [17]. Results depict that ChIAs have higher sensitivity but are not of very good specificity, resulting in higher false positive screening results in serological HIV screening. False-positive results could cause considerable unnecessary apprehension in patients when used diagnostically. Besides, following up on false results will consume time and money. Many developing countries cannot afford to go for so many costlier and labor intensive diagnostic methods like Western blot and PCR assays due to higher false-positive rate of 4th screening assays. In another word, nearly 3 times more samples would need repeated testing. However, in developing countries, such as China, it was hard for a laboratory to afford the burden for so many false-positive samples. China has a large population, considerable diversities exist in diagnosis of HIV/AIDS at the levels of township, county, province and medical university/college hospitals. As a developing country, China has put the control and prevention of HIV/AIDS as the most important issue in its public health care. HIV infection is becoming a chronic disease in increasing numbers of survivors, requiring organized medicine more than ever but limited medical resources may still remain a barrier of effective health care. If widespread use of routine screening could offer benefits at a reasonable cost, the potential cost-savings are remarkable. The development and affordability of reliable and less expensive serological testing assays continue to be challenges in our settings. Though national and provincial level support has been provided to build local capacity in areas affected by HIV,many key challenges and disputes remain in promoting universal access to HIV diagnosis, treatment and care and support [18], [19]. The switch from the 3rd generation to 4th generation assay will lead to a decrease in specificity and to more specimens with discordant results between the screening assay and Western blot confirmation. These data are important to consider as local, regional, and national laboratories make decisions about what HIV testing algorithm is most appropriate for the populations they serve.

Table 2 and 3 indicated that PPV, Sp and Se of EIA were satisfactory in this setting. The repeatability and interpersonal variation was also good with this EIA assay (Table 2 and 4). With the availability of EIA commercial tests, EIA has become possible to cost-effectively screen smaller large of specimens without a huge initial input for the laboratory. The EIA could be suitable for initial screening in the HIV testing algorithm in resource-limited settings where the laboratory infrastructure is neither present nor well-outfitted [20]. In addition, facilities for 4th generation HIV tests, such as ChIA tests, are unavailable in smaller towns and rural areas of China. The format of an EIA is the most appropriate for screening large numbers of specimens with limited infrastructure due to its high specificity and sensitivity.

The majority of western blot-discordant samples turned negative. Only 4 samples not initially meeting criteria for positivity turned clear-cut positive using ChIA (Table 5). Most weakly reactive or borderline results were negative when determined by WB (western blot) and RNA tests. All cases with COI ratio (ChIA) <15.0 were false-positive, reflecting the unnecessarily frequent laboratory testing to carry out redetermination, especially in weakly reactive samples. In the ChIA assay, only 2 (2.2%) of 89 cases with COI ratio of <50 had HIV infection (Table 7). To some degree, health resources were wasted due to the higher false positive rate of ChIA. Table 7 also shows a clear relation between the quantitative assay values and positive predictive value. On the basis of these data, we suggest that caution be used in interpreting results at the low end of the distribution of positive values for both assays. P24 and gp 160 bands are often present on indeterminate WB and can be an indication of false positive results (Table 6), however, they are also the first bands to evolve on WB during seroconversion, so care should be taken in interpreting these as likely false positive results [21]. Besides, the potential sources of false positivity also include sample handling and laboratory practice, such as sample contamination and sample mislabeling.

Relative to the magnitude of the epidemic, government funds available for HIV prevention are scarce. To optimize use of funds, emphasis should be placed on cost-effective strategies, instead of expanding the use of strategies that are inherently limited or costly. In these resource-limited settings, the use of WB and PCR due to the false-positive results has limited the expansion of testing programs. The benefit of an HIV combination assay in urban centers with high sample throughput was described in an earlier study [22]. To be useful, diagnostic methods must be accurate, simple and affordable for limiting the spread of infection and for the appropriate clinical management of persons infected with HIV [23]. It is imperative that these new diagnostics, like all the other tests, are rigorously and properly evaluated in the situations in which they will be deployed in disease control before they are released for general use. Although 4th generation HIV tests offer the great advantages of improved precision, reliability, technical simplicity, short turn-around time, high-speed throughput, and full automation, particularly for high-volume hospital laboratories,they are primarily intended for advanced diagnostic laboratories, and may not address testing methods or strategies in more resource-limited settings where lack trained personnel. In many rural areas where most of China’s HIV-positive population resides, the physical infrastructure exists, but the staffs do not have the skills or reagents to use it [24]. The combination of insufficiently trained staffs, inadequate technical resources, and a largely remote, poorly educated, rural population represents a challenge to the implementation of 4th generation HIV tests. Besides, adding p24 antigen, RNA tests to the current only antibody screening cannot be regarded as a cost-effective health-care intervention for developing countries [25].

In the field of the diagnostic monitoring of the clinical management of infectious diseases a necessary cost-saving can be achieved by apt services that use less expensive and better coordinated modern technology with a significantly improved quality. Well-organized regional diagnostic service provisions should be better supported by avoiding a scenario when limited medical resources are wasted on expensive and/or untried methods without a careful constructive scrutiny [26], [27], [28]. Similarly, a more flexible design with the full involvement of local doctors and healthcare personnel should lead to the reduction of unnecessarily frequent and/or over-ambitious laboratory testing. This policy would give more chance to a fuller utilization of the available accurate diagnostic results and could also be pioneered for the resource-limited areas. Success to implement routine screening at reasonable cost represents a significant financial assistance to patients who are currently infected, those at risk for infection, and the future health of the nation.

This study has two limitations. Firstly, the same specimens were not tested with both assays over the different time periods. However, specimens (hospitalized patients, outpatients, pre-surgery, health care workers and anonymous testing) are transmitted to our laboratory randomly. In addition, the study has a large enough “n” to conduct applicable statistical comparisons. As a result, we can assume that the patient populations tested over the different time periods were randomly assigned. Besides, 3740 samples on paired testing (Table 2) and 829 HIV-1 infected samples (Table 3) are tested on each assay, which would strengthen the statistics. Secondly, specimens which were not taken through the entire algorithm (i.e., repeatedly reactive with either screening assay but subsequently not tested by Western Blot) have been removed from study analyses. The cause always include the factor of death,losing contact and unwillingness. Therefore, a separate computer-based storage and retrieval of results were very helpful in ascertaining proper follow-up of discordant results, especially for labs handling a large number of specimens.

### Conclusion

Caution is needed before shifting from 3rd to 4th generation HIV tests. Since none of these tests are perfect, different geographic and ethnic area probably require different considerations with regard to HIV testing methods, taking into account the local conditions. Laboratories should provide different levels of services, based on mission, the spectrum of infections encountered, financial resources and the population of patients served.

## Supporting Information

Supplementary Materials S1 (DOC)
