# Supplementary material for: Comparative Performance of Electrochemiluminescence Immunoassay and EIA for HIV Screening in a Multiethnic Region of China
Source: PLoS One. 2012 Oct 29;7(10):e48162. doi: 10.1371/journal.pone.0048162 (PMC3483174; doi:10.1371/journal.pone.0048162)
Supplement: Supplementary Materials S1 — (DOC) [file pone.0048162.s001.doc]

**Supplementary Materials**

The characteristics of the reagents and the technical specifications of each instrument are available at:

<http://www.fda.gov/downloads/BiologicsBloodVaccines/BloodBloodProducts/ApprovedProducts/PremarketApprovalsPMAs/ucm092879.pdf>

(COBAS AmpliPrep/COBAS TapMan HIV-1 Test)

<http://rochecanada.com/fmfiles/re7234008/package_inserts/HIVCOMBI-04860446190-ENGLISH-V5-CAN.pdf>(Elecsys HIVcombi assay)

<http://www.diagnostictechnology.com.au/persistent/catalogue_files/products/MPBio-HIV-p.pdf> (Western Blot HIV blot 2.2)

<http://www.livzondiagnostics.com/Product_show.asp?id=36>(Anti-HIV ELISA kit)
